# Identification of two molecular subtypes and a novel prognostic model of lung adenocarcinoma based on a cuproptosis-associated gene signature

**DOI:** 10.3389/fgene.2022.1039983

**Published:** 2023-01-12

**Authors:** Jinlin Zhou, Dehe Chen, Shiguo Zhang, Chunmei Wang, Li Zhang

**Affiliations:** ^1^ Department of Respiratory Medicine, Bazhong Central Hospital, Bazhong, Sichuan, China; ^2^ Department of Medical Oncology, Chongqing University Cancer Hospital, Chongqing, China

**Keywords:** cuproptosis, LASSO, prognostic model, CIBERSORT, immune microenviroment

## Abstract

Lung adenocarcinoma is the most common subtype of lung cancer clinically, with high mortality and poor prognosis. Cuproptosis present a newly discovered mode of cell death characterized by aggregation of fatty acylated proteins, depletion of iron-sulfur clusterin, triggering of HSP70, and induction of intracellular toxic oxidative stress. However, the impact of cuproptosis on lung adenocarcinoma development, prognosis, and treatment has not been elucidated. By systematically analyzing the genetic alterations of 10 cuproptosis-related genes in lung adenocarcinoma, we found that CDKN2A, DLAT, LIAS, PDHA1, FDX1, GLS, and MTF1 were differentially expressed between lung cancer tissues and adjacent tissues. Based on the expression levels of 10 cuproptosis-related genes, we classified lung adenocarcinoma patients into two molecular subtypes using the Consensus clustering method, of which subtype 2 had a worse prognosis. Differential expression genes associated with prognosis between the two subtypes were obtained by differential analysis and survival analysis, and cox lasso regression was applied to construct a cuproptosis-related prognostic model. Its survival predicting ability was validated in three extrinsic validation cohorts. The results of multivariate cox analysis indicated that cuproptosis risk score was an independent prognostic predictor, and the mixed model formed by cupproptosis prognostic model combined with stage had more robust prognostic prediction accuracy. We found the differences in cell cycle, mitosis, and p53 signaling pathways between high- and low-risk groups according to GO and KEGG enrichment analysis. The results of immune microenvironment analysis showed that the enrichment score of activated dendritic cells, mast cells, and type 2 interferon response were down-regulated in the high-risk group, while the fraction of neutrophils and M0 macrophages were upregulated in the high-risk group. Compared with the high-risk group, subjects in the low-risk group had higher Immunophenoscore and may be more sensitive to immunotherapy. We identified seven chemotherapy agents may improve the curative effect in LUAD samples with higher risk score. Overall, we discovered that cuproptosis is closely related to the occurrence, prognosis, and treatment of lung adenocarcinoma. The cuproptosis prognostic model is a potential prognostic predictor and may provide new strategies for precision therapy in lung adenocarcinoma.

## 1 Background

Lung adenocarcinoma (LUAD), a primary pathological type of lung cancer, was characterized by high mortality and poor prognosis (Duma et al., 2019). Although the advent of immunotherapy and targeted therapy has led to new advances in treating lung adenocarcinoma, its 5-year overall survival rate is less than 20% (Lin et al., 2016). Mining new biomarkers to predict the prognosis of patients with lung adenocarcinoma is imminent.

Copper can participate in the occurrence and development of malignant tumors due to its roles in enhancing angiogenesis, cell proliferation, and metastasis (Theophanides and Anastassopoulou, 2002; Park et al., 2016). The latest research uncovers a novel regulatory mechanism of cell death named cuproptosis. Cuproptosis mainly occur in cells dependent on respiration and the TCA cycle. In the cuproptosis process, promoting the binding of copper to fatty acylated components leads to cascade including aggregation of fatty acylated proteins, iron-sulfur cluster proteins exhaustion, the trigger of HSP70, induction of intracellular Toxic oxidative stress, ultimately causing cell death (Tsvetkov et al., 2022). Studies have demonstrated that copper is abundant in lung cancer patient’s serum or tumor tissues. In addition, copper is associated with tumorigenesis, invasion, and metastasis (Wang et al., 2021). However, few reports involve the regulatory mechanism of cuproptosis on lung adenocarcinoma. In this paper, we clustered lung adenocarcinoma patients into two subtypes based on the mRNA expression of 10 copper death-related genes by consensus clustering. A prognostic model was constructed based on differential genes between the two subtypes, and its underlying immune mechanism was explored.

## 2 Materials and methods

### 2.1 Data collection and processing

Ten cuproptosis-related genes were obtained from the science journal article (Tsvetkov et al., 2022). Lung adenocarcinoma transcriptome data and clinical data were acquired from TCGA project (http://xena.ucsc.edu/) and GEO (https://www.ncbi.nlm.nih.gov/gds), and TCGA transcriptome data with FPKM format (fragments per kilobase per million mapped reads) was downloaded, which was further converted to TPM (transcripts per million) enhancing the comparability with the microarray data. The CNV (copy number variation), SNP (single nucleotide polymorphism), and drug response analysis results of lung cancer patients in TCGA were achieved from the GSCA website (http://bioinfo.life.hust.edu.cn/web/GSCALite/) (Liu et al., 2018). The drug sensitivities were calculated using the training data from the Cancer Therapeutics Response Portal (CTRP) (Basu et al., 2013). We compare the mRNA expression of 10 cuproptosis-related genes between 526 tumor subjects and 59 tumor-adjacent subjects in TCGA. GEO microarray transcriptome data were normalized using the normalizeBetweenArrays function of the “limma” package (Ritchie et al., 2015). Log2 transformed all gene expression data. In this study, 1417 LUAD patients and six LUAD cohorts were enrolled including TCGA (503), GSE30219 (85), GSE50081 (127), GSE72094 (398), GSE31210 (246), and GSE3141 (58). The clinical characteristics of the six cohorts were represented in the Supplementary Table S1. Three cohorts, including TCGA, GSE30219, and GSE50081, were merged as a pooled dataset with 738 LUAD patients. The “combat” function of the SVA package (Leek et al., 2012) is used to remove the batch effect. Besides, GSE32863 cohort containing transcriptome data of 58 lung adenocarcinoma and 58 adjacent non-tumor lung fresh frozen tissues, was used to verify the expression of ten cuproptosis related genes and prognostic signature genes (20 of 22 signature genes available due to GPRIN1, HJURP unavailable).

### 2.2 Consensus clustering

Lung cancer samples in the pooled dataset were grouped according to the mRNA expression of 10 cuproptosis-related genes using a consensus clustering method. “Cancer Subtypes” package (Xu et al., 2017) was applied for the consensus clustering analysis and comparing the prognostic difference between two clusters. “Limma” package was used to analyze the differentially expressed genes between the two subtype groups. Those differential expression genes were defined as cuproptosis pattern differentially genes (CPDGs). The screening criteria were FDR <.05 and log|FC| > .5.

### 2.3 Construction and evaluation of prognostic model

In the pooled dataset, univariate cox regression analysis was used to find out prognosis-related CPDGs, whose gene expression values and overall survival data were further input to construct a cuproptosis prognostic model using cox least absolute shrinkage and selection operator (lasso) regression analysis. The pooled dataset was used as the training set, while the other three cohorts, including GSE72094, GSE31210, and GSE3141, were validation sets. The risk scores were calculated according to the below formula.

RiskScores=∑coefficient∗GeneExpression

The median of risk scores was regarded as the cut-off to divide the LUAD samples into a high and low-risk groups. The distribution of risk scores between different clinical subgroups was compared in training and test cohorts. Due to unavailable clinical data, GSE3141 wasn’t included. Kaplan-Meier (KM) survival curves were drawn for LUAD subjects in the high and low-risk groups. The AUC (area under the curve) value of timeROC (time receiver operating characteristic) curve was used to evaluate the survival prediction ability of 1, 2, and 3 year of overall survival (OS) time. Besides, multivariate cox analysis was applied to demonstrate whether the cuproptosis prognostic model is an independent survival predictor in training and test sets. The GSE3141 cohort was excluded for its unavailable clinical data.

### 2.4 Nomogram drawing

The risk score of the cuproptosis prognostic model was integrated with clinical features such as age, gender, and stage to fabricate a nomogram (Iasonos et al., 2008) using rms package. TimeROC and calibration curves were used to evaluate the survival prediction ability of the mixed model.

### 2.5 Gene ontology (GO) and kyoto encyclopedia of genes and genomes (KEGG) enrichment analysis

Limma package was utilized to screen the differential expression genes between high and low-risk groups. The selecting criteria were FDR <.05 and log|fc| >1. Afterward, Gene Ontology (GO) (Gene Ontology Consortium, 2015) and Kyoto Encyclopedia of Genes and Genomes (KEGG) (Kanehisa and Goto, 2000) enrichment analysis were performed for the differential expression genes.

### 2.6 Immune infiltration analysis

The immune cell fraction of each LUAD sample was calculated by the CIBERSORT algorithm of “IOBR” R package, which also was applied to analyze the Immunophenoscore (IPS) characterizing the immunogenicity of LUAD samples (Newman et al., 2015; Charoentong et al., 2017; Zeng et al., 2021). In addition, the single sample gene set enrichment analysis (ssGSEA) (Barbie et al., 2009) was used to measure the relative abundance level of 16 immune cells and 13 immune function signatures in LUAD samples.

### 2.7 Drug sensitivity analysis

Oncopredict package was used to calculate the estimation IC50 for each sample based on ridge regression method (Maeser et al., 2021). The drug sensitivity and transcriptome data of cell lines in Genomicsof Drug Sensitivity in Cancer (GDSC) database was regarded as the training data. (Yang et al., 2013) considering the low sample size of GSE3141, we included the merge cohort, GSE31210, and GSE72094 for drug sensitivity analysis. The correlation coefficients were obtained by conducting pearson correlation analysis between drug sensitivities and the risk scores. To obtain robust result, we included the drugs whose estimation IC50 values correlated with risk scores in all three cohorts simultaneously. FDR<.05 was considered significant.

### 2.8 Statistical analysis

R language (version 4.0.5) was primarily used for all data analysis in the study. The “ggplot2” package was applied for figure fabrication. Wilcox or Kruskal-Wallis test was used to estimate the statistical significance of quantitative data when comparing two or more types. For Kaplan-Meier survival analysis, the log-rank method was used to test the statistical significance. Cox lasso regression analysis was conducted using the R package “glmnet”. *p* < .05 was considered statistically significant. The Benjamini-Hochberg method was applied to limit the false discovery rate (FDR) when conducting multiple hypothesis testing.

## 3 Result

### 3.1 Transcriptome and genomic variance of ten cuproptosis-related genes in LUAD

**FIGURE 1:**
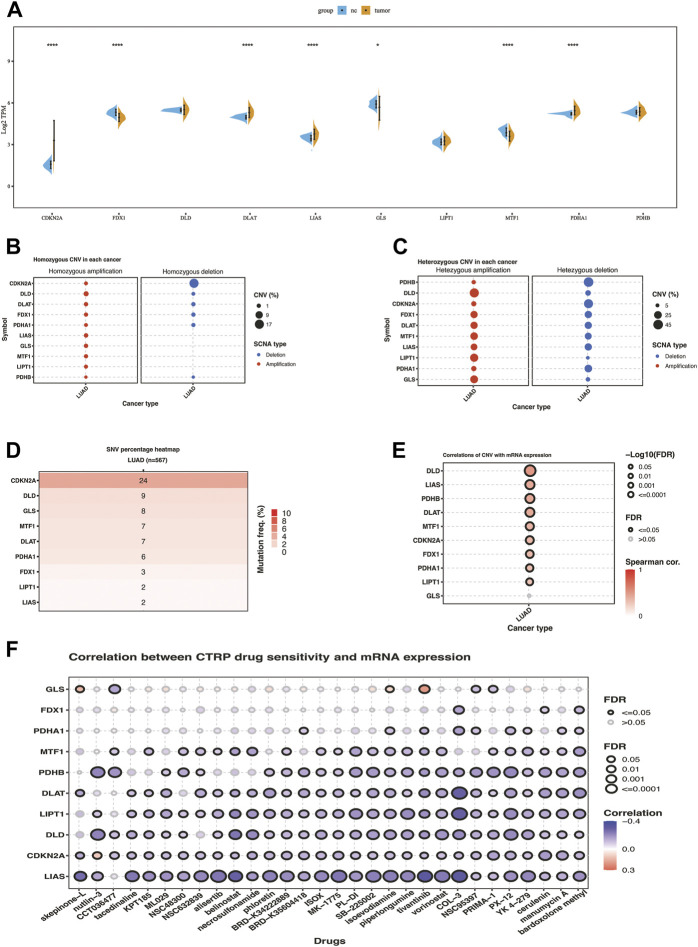
Transcriptome and genomic variation of 10 cuproptosis-related genes in TCGA-LUAD. **(A)** the gene expression difference of 10 cuproptosis genes between NC and lung cancer in TCGA. **(B)** Homozygous CNV of 10 cuproptosis-related genes in LUAD. **(C)** Heterozygous CNV of 10 cuproptosis-related genes in LUAD. **(D)**SNV percentage heatmap of 10 cuproptosis genes in LUAD. **(E)** Correlations of CNV of ten cuproptosis-related genes with mRNA expression in LUAD. **(F)** Correlation between CTRP drug sensitivity and mRNA expression of ten cuproptosis-related genes. The symbols *, **, ***, *** corresponded to *p* < .05, .01, .001, .0001, respectively. *p*< .05 was regarded as significant.

Differential expression analyses of ten cuproptosis-related genes between LUAD and tumor-adjacent samples were conducted (Figure 1A). Among them, the significantly upregulated genes in LUAD were CDKN2A, DLAT, LIAS, and PDHA1, while FDX1, GLS, and MTF1 were downregulated. Similar expression pattern was found in cohort GSE32863 except for the MTF1 gene, which exhibited significantly higher expression in lung cancer tissues compared with normal tissues (Supplementary Figure S1A). SNV and CNV analysis showed that some cuproptosis-related genes exhibited genomic variance. The mutation frequency of CDKN2A was 24%, far more than the others. The homozygous deletion frequency of CDKN2A was much higher than homozygous amplification frequency. In heterozygous CNV analysis, DLD, LIPT1, and GLS were prone to heterozygous amplification, while PDHB, CDKN2A, and PDHA1 were prone to heterozygous deletion (Figures 1B–D). The correlation of CNV with mRNA expression showed that copy number increasing of cuproptosis-related genes significantly enhanced the mRNA expression except for the GLS (Figure 1E). We also correlated drug sensitivity and cuproptosis-related mRNA expression according to the Cancer Therapeutics Response Portal (CTRP) database. The bubble chart showed that the mRNA expression of ten cuproptosis-related genes was negatively correlated to the IC50 value of cancer therapy agents (Figure 1F). We used the best cut-off to compare KM overall survival time between high and low gene expression groups of ten cuproptosis-related genes. We found that seven genes significantly correlated with prognosis, where PDHA1, DLAT, DLD, and CDKN2A were high-risk factors for the prognosis of LUAD patients in the TCGA cohort, while MTF1, LIPT1, and GLS were favorable factors (Supplementary Figure S2).

### 3.2 Identification of two cuproptosis-related patterns in LUAD

**FIGURE 2:**
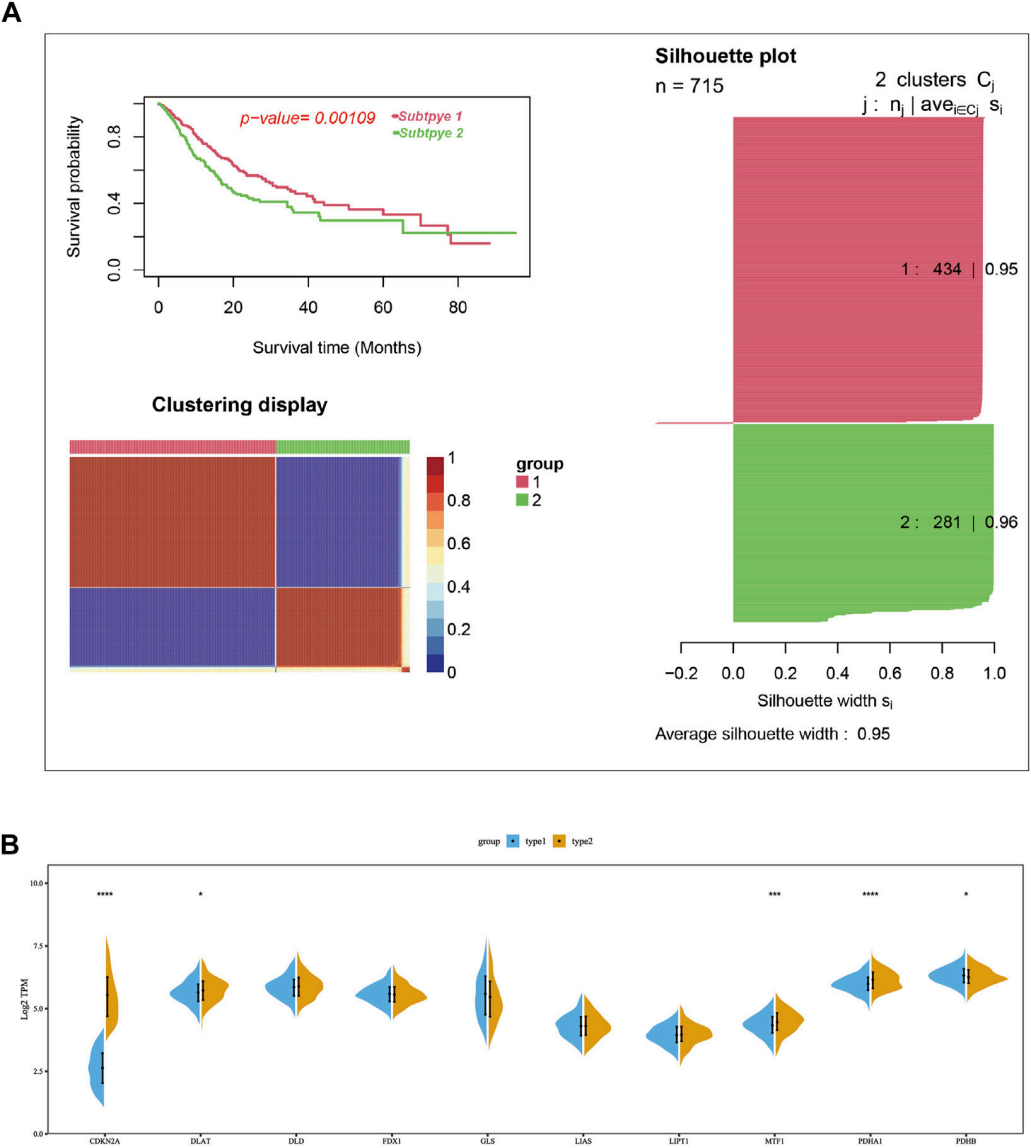
Two cuproptosis-related subtypes of LUAD were identified using the consensus cluster method. **(A)** Two LUAD subtypes were obtained according to the mRNA expression of cuproptosis-related genes using the consensus clustering in the merge cohort, in which subtype 1 had a poorer prognosis compared with sub type 2. **(B)** The mRNA expression difference of ten cuproptosis-related genes between two subtypes in merge cohort. In the silhouette plot of Figure 2A, each row represent the Silhouette Coefficient (range from 0–1) of each sample, higher Silhouette Coefficient means better clustering effect, the average Silhouette Coefficient for subtype 1 and 2 were .95 and .96. The symbols *, **, ***, *** corresponded to *p* < .05, .01, .001, .0001, respectively. *p* < .05 was regarded as significant.

Consensus clustering was used to group the lung cancer samples of the pooled dataset based on the mRNA expression of ten cuproptosis-related genes (Supplementary Table S2). Since the CDF curve of cluster number two was smoothest with average silhouette width of .95, we grouped the samples into two subtypes. KM overall survival analysis showed that patients in subtype2 have a poorer prognosis than subtype1 (Figure 2A). Differential expression analysis results showed that the mRNA expression of CDKN2A, DLAT, PDHA1, and MTF1 were significantly upregulated in subtype 2, while PDHB was upregulated in subtype1 (Figure 2B).

### 3.3 Prognostic model construction and evaluation

**FIGURE 3:**
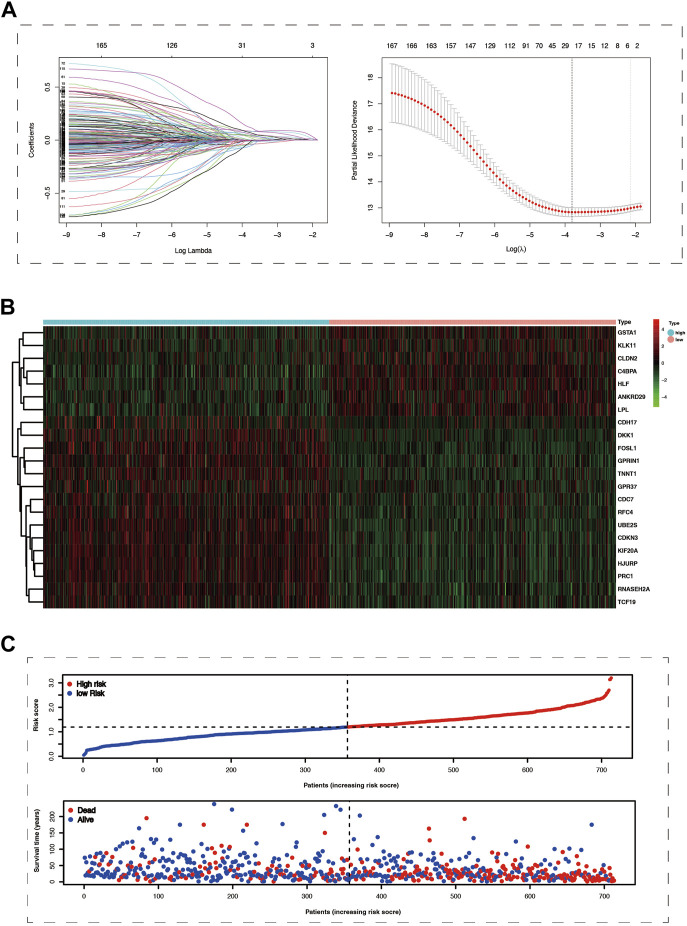
The Prognostic model was constructed using cox lasso method in the merge cohort. **(A)** The left part of picture showed the coefficients of 169 survive-related genes dropping speed as the log lambda increased. The right section of the figure exhibited that partial likelihood deviance was applied to evaluate the fitness of the prognostic model, the minimum value among which would correspond to a specific log lambda. **(B)** The heat map showed the mRNA expression of 22 prognostic model genes in the high and low-risk groups. **(C)** The more significant number of deaths and lower survival time were represented in the high-risk group.

Conducting differential expression analysis between subtype 1 and subtype 2 generated 235 CPDGs in the pooled dataset (Supplementary Table S3), among which 169 CPDGs significantly correlated with the prognosis according to the univariate cox regression analysis result (Supplementary Table S4). Next, we conducted LASSO Cox regression analysis on the 169 OS-related CPDGs using one standard error (SE) and 10-fold cross-validation, and a 22-genes cuproptosis prognostic signature was formed in pooled dataset regarded as the training cohort (Figures 3A). The signature consists of 22 CPDGs, including ANKRD29, C4BPA, CDC7, CDH17, CDKN3, CLDN2, DKK1, FOSL1, GPR37, GPRIN1, GSTA1, HJURP, HLF, KIF20A, KLK11, LPL, PRC1, RFC4, RNASEH2A, TCF19, TNNT1, and UBE2S, whose coefficients were presented in Supplementary Table S5. Among the 22 CPDGs, seven genes including KLK11, LPL, CLDN2, C4BPA, GSTA1, ANKRD29, and HLF were favorable prognostic factors and the rest of them were high risk factors according to the univariate cox regression analysis result. Then we investigated the transcriptome expression difference of those genes between lung cancer and normal tissues. In TCGA cohort, 19 of the 22 CPDGs showed significant expression difference except the three genes including CLDN2, DKK1, and FOSL1. Among the 19 significant CPDGs, low risk genes were upregulated in normal tissues while high risk genes show higher expression in lung cancer tissues. Above transcriptome differences were also verified in GSE32863 (Supplementary Figures S1B,C). The risk score of each sample was calculated using the formula of method section (Supplementary Tables S6–S9), and LUAD samples were stratified into high-risk group (*n* = 356) and low-risk group (*n* = 357) based on the median of risk scores in the pooled cohort. Not only in the training set but also in three test sets (GSE72094, GSE31210, and GSE3141), risk scores exhibited similar distribution, and samples with higher risk scores corresponded to shorter OS time and more death, the expression characteristics of 22 CPDGs between high and low-risk groups were similar as well (Figures 3B,C; Supplementary Figure S3).

**FIGURE 4:**
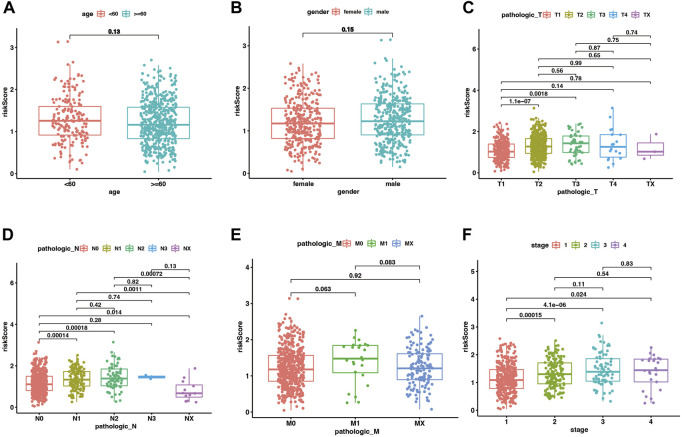
The distribution of risk scores between different clinical subgroups in the merge cohort. **(A–F)** corresponded to two age groups (<60 vs. ≥ 60 years), gender (male vs. female), Tumor classification, node classification, metastasis classification, and stages. *p* < .05 was considered significant.

In the training set of the pooled dataset, there was no noticeable difference in risk score distribution neither between two age groups (≥60 years vs. <60 years) nor two gender types (Male vs. Female), while male LUAD patients own significant higher risk score in GSE72094 cohort. Generally, higher TNM (Tumor classification, node classification, metastasis classification) and stages go along with higher risk scores, demonstrated in both the training and validation sets (Figure 4; Supplementary Figure S4).

**FIGURE 5:**
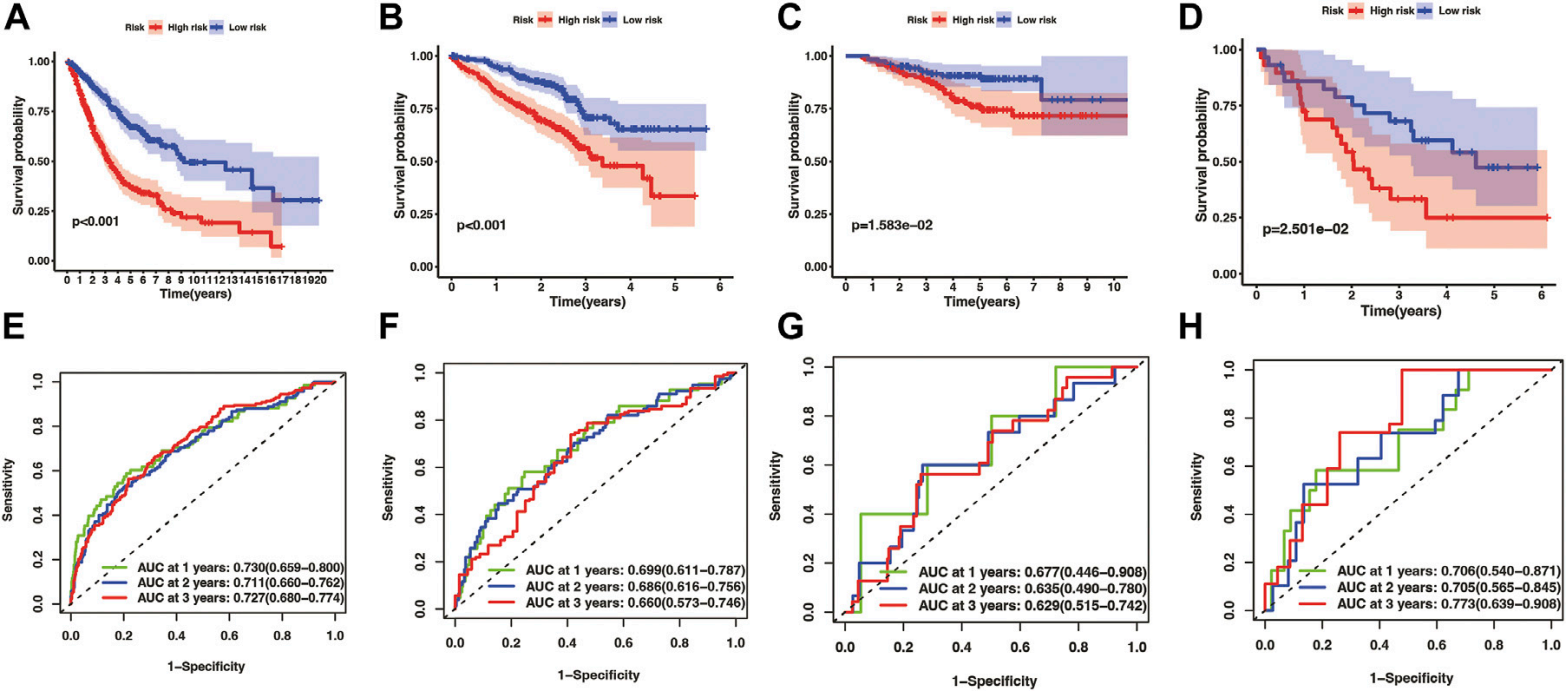
Evaluation of prognostic model in training and test cohorts, including merge cohort **(A,E)**, GSE72094 **(B,F)**, GSE31210 **(C,G)**, and GSE3141 **(D,H)**. The overall survival KM curves showed that LUAD patients in the high-risk group had a shorter OS time comparing with the low-risk group **(A–D)** pictures from **(E–H)** were timeROC curves of 1, 2, and 3 year overall survival time, which showed that the prognostic model had an excellent 1, 2, and 3 year OS time predicting ability.

Kaplan-Meier analysis manifested that the patients in the high-risk group had shorter OS time than the low-risk group in both the training cohort and three test cohort (pooled dataset, *p* < .001; GSE72094, *p* < .001; GSE31210, *p* = 1.583e−02; GSE3141, *p* = 2.501e−02) (Figures 5A–D). The timeROC curves of the risk score also exhibit good OS prediction ability in both training and test set (Figures 5E–H), especially at 1 year overall survival time [AUC (95% CI): pooled dataset, .730(.659–.800); GSE72094, .699(.611–.787); GSE31210, .677(.446–.908); GSE3141, .706(.540–.871)].

### 3.4 The 22-genes cuproptosis signature was an independent prognostic factor

**FIGURE 6:**
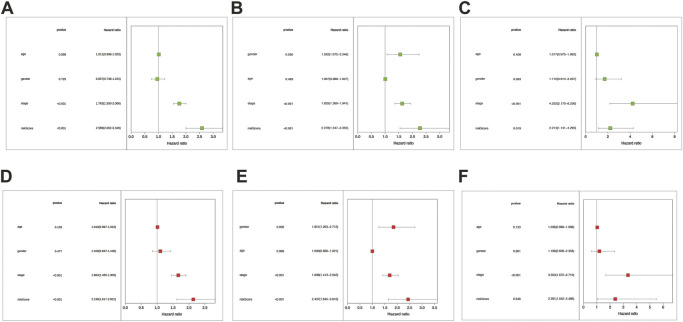
The risk score was the independent prognostic factor in LUAD cohorts. Forest maps were made based on the univariate cox analysis results **(A–C)**. Multivariate cox analysis results were shown in pictures **(D–F)**. **(A,D)** correspond to the merge cohort; **(B,E)** correspond to the GSE72094 set; **(C,F)** correspond to the GSE31210 set. *p* < .05 was considered significant.

We conducted univariate and multivariate Cox regression analyses among the retrievable variables to identify whether the risk score could predict the OS independently. Due to the non-availability of clinical data in the GSE3141 cohort, the univariate and multivariate cox analyses were not carried out in the GSE3141 cohort. In univariate Cox analyses, the risk score was significantly related with the overall survival in both the training and the validation cohort [pooled dataset: 2.589 (2.002–3.349) HR (95% CI), *p* < .001; GSE72094: 2.278(1.547–3.355), *p* < .001; GSE31210: 2.214(1.141–4.295), *p* = 0.019] (Figures 6A–C). After rectification for other noise factors, the risk score was demonstrated to be an independent prognostic factor in the multivariate Cox regression analysis (pooled dataset: 2.136(1.617–2.821) HR (95% CI), *p* < .001; GSE72094: 2.437(1.645–3.610), *p* < 0.001; GSE31210: 2.391(1.042–5.486), *p* = .04; Figures 6D–F).

### 3.5 Mixed-model strengthened the accuracy of OS prediction

**FIGURE 7:**
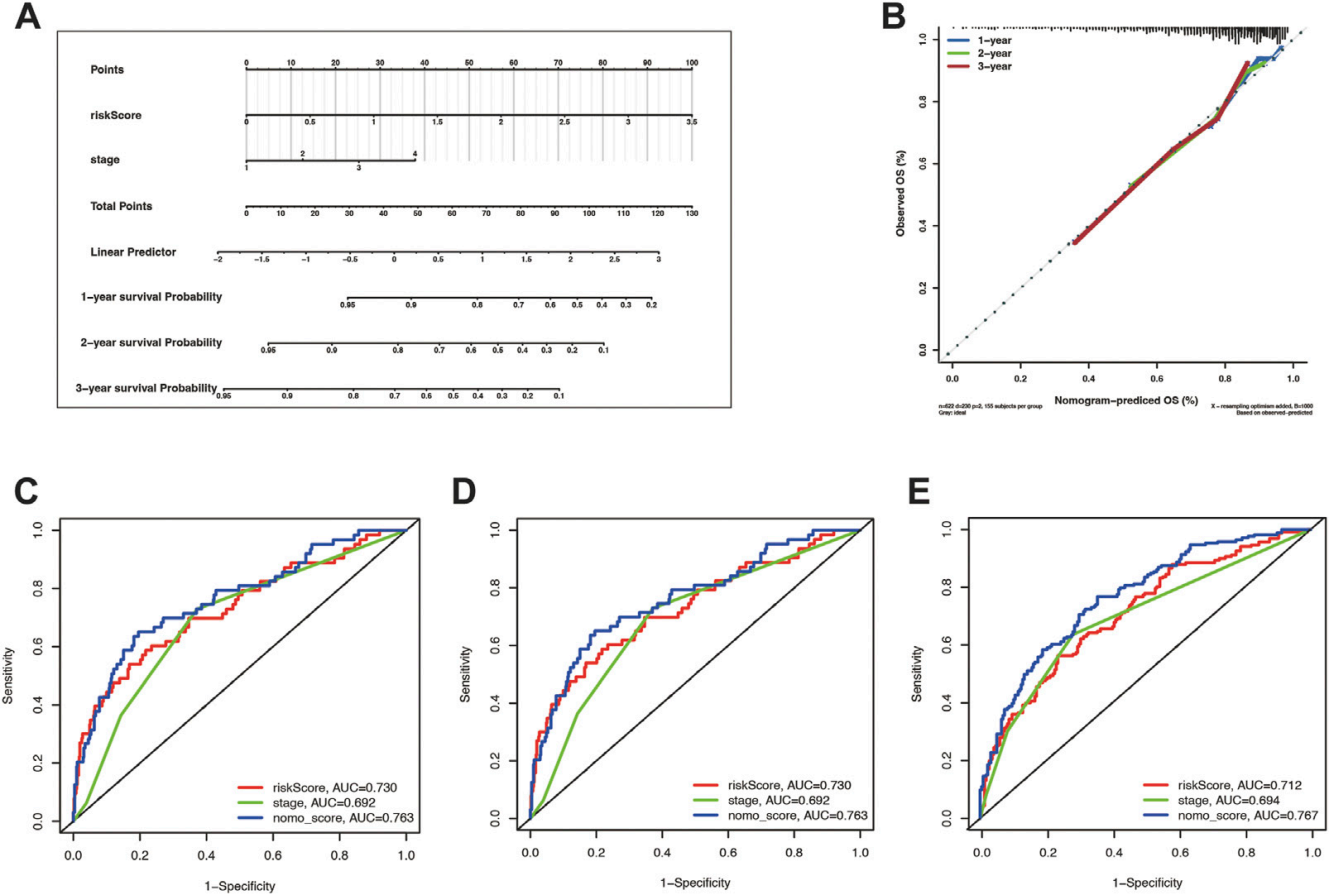
The risk score combining stage showed a better prognostic prediction power. **(A)** Nomogram with a total score of risk score and stage predicting the overall survival time was drawn in the merge cohort. **(B)** The calibration curve showed the difference extent between nomogram-predicted OS and observed OS. **(C–E)** were the timeROCs for features including risk score, stage, and nomo_score in 1, 2, and 3 year overall survival time, respectively.

Tumor staging can describe the severity and involvement range of malignancy and predict the prognosis of patients. We hoped to explore whether tumor staging combined with the cuproptosis prognostic model could more accurately predict the OS of lung cancer patients. To this end, we draw a nomogram based on the Cox regression model (Figure 7A). The calibration curves illustrate the high accuracy of the total score of the staging and the cuproptosis prognostic models in predicting 1, 2, and 3-year overall survival (Figure 7B). What’s more, through the timeROC curve, we found that the AUC value of the mixed model combining the stage and the cuproptosis prognostic model to predict the 1, 2, and 3-year overall survival was higher than that of them alone, indicating that the stage combined with the cuproptosis prognostic model can more accurately predict the overall survival (Figures 7C–E).

### 3.6 GO and KEGG enrichment analysis

**FIGURE 8:**
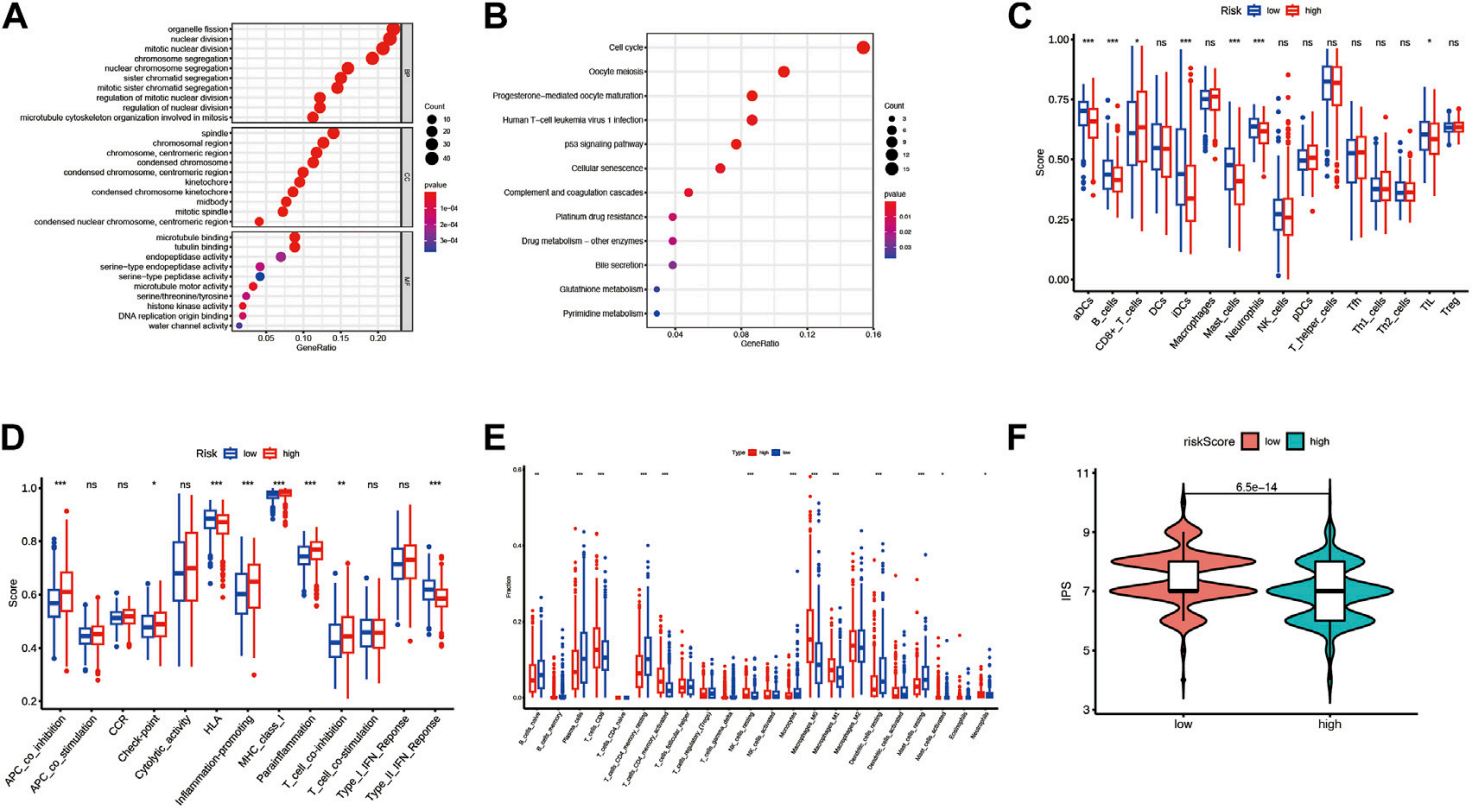
The differences in the immune environment between the high and low-risk groups of the merge cohort. **(A)** The GO enrichment result for differential expression genes between the high and low-risk group. **(B)** The KEGG enrichment result for differential expression genes between the high and low-risk group. **(C)** 16 immune cell signatures were compared between the high and low-risk groups. **(D)** 13 immune function signatures were compared between the high and low-risk group I **(E)** immune cell fractions inferred by CIRBERSORT were compared between the high and low-risk groups. **(F)** Immunephenoscore (IPS) was compared between high and low-risk groups. The symbols *, **, ***, *** corresponded to *p* < .05, .01, .001, .0001, respectively. *p* < .05 was regarded as significant.

To investigate the risk score related biological functions and pathways, GO and KEGG enrichment analyses were conducted for differential expression genes between the high-risk and low-risk groups. Combining GO and KEGG-enriched results in four cohorts (pooled dataset, GSE72094, GSE31210, and GSE3141), we found that DEGs were mainly associated with cell cycle, mitosis, p53 signaling, complement and coagulation cascades (Figures 8A,B; Supplementary Figure S5; Supplementary Tables S10–S13).

### 3.7 Immune microenvironment analysis

To investigate whether risk scores are associated with immune infiltration, we used two methods to quantify immune cells or immune function in each sample: CIRBERSORT and ssGSEA, and compared them between high-risk and low-risk groups. For the sake of rigor, the simultaneous presence of statistical significance in the four arrays was considered significant. In the ssGSEA analysis results, the enriched scores of activated dendritic cells, mast cells, and Type II interferon Reponse were significantly down-regulated in the high-risk group compared to the low-risk group (Figures 8C,D; Supplementary Figure S6). By CIRBERSORT analysis, we found that the fraction of neutrophils and M0 macrophages were significantly higher in the high-risk group than in the low-risk group (Figure 8E; Supplementary Figures S7A–C). In addition, patients in the high-risk group had lower IPS than those in the low-risk group significantly except for the patients in the GSE3141 cohort (Figure 8F; Supplementary Figures S7D–F).

### 3.8 Drug sensitivity analysis

**FIGURE 9:**
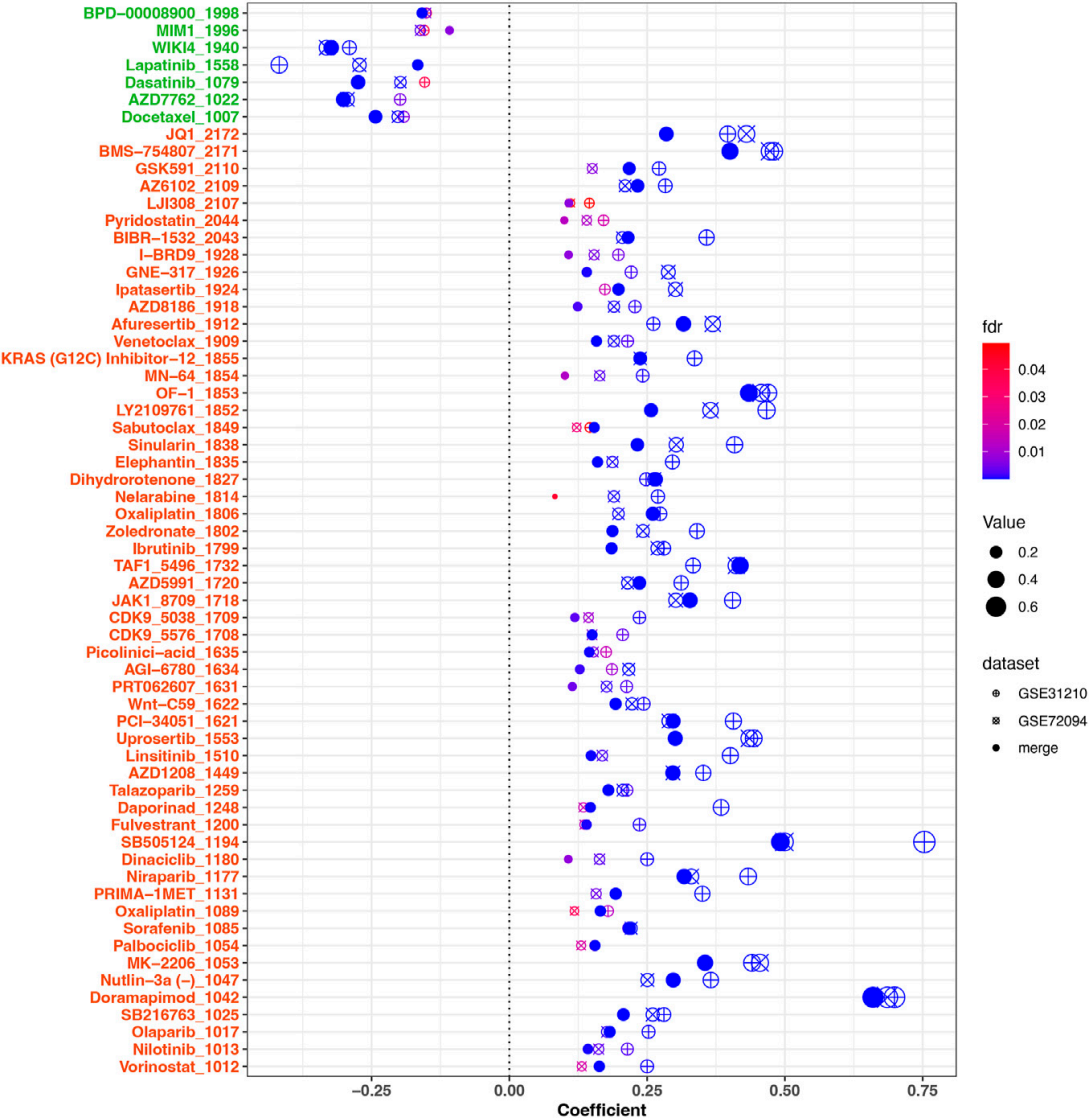
Correlation analysis between drug sensitivities of agents and risk scores. 61 significant agents were represented in the figure. The horizontal ordinate was the correlation coefficient, while the ordinate represents the name of the drugs. Different shapes of dots represent different cohorts. The larger the dot, the greater the absolute value of the correlation coefficient. The color corresponded to the FDR value. On the ordinate, green marks negatively correlated drugs, while red marks positively correlated drugs. FDR <.05 was considered significant.

To investigate the relationship between the risk scores and drug sensitivities, we calculated the estimation IC50 value for lung cancer samples based on the drug sensitivity data in GDSC. Risk score related drugs were obtained in three cohorts (merge cohort, GSE31210, GSE72094). The result represented in Figure 9. A total of 61 agents were found significantly correlated with risk scores. Among them, 54 agents showed lower drug sensitivities in the higher risk samples, most of which target PI3K/MTOR signaling (7 agents: MK-2206, Uprosertib, Afuresertib, AZD8186, Ipatasertib, GNE-317, and LJI308), Genome integrity (4 agents: Olaparib, Niraparib, Talazoparib, and BIBR-1532), Chromatin histone methylation (4 agents: GSK591, Vorinostat, PCI-34051, and OF-1), Cell cycle (4 agents: Palbociclib, Dinaciclib, CDK9_5576, and CDK9_5038), and kinases (4 agents: Sorafenib, AZD1208, PRT062607, JAK1_8709, and Ibrutinib), while seven agents exhibited higher drug sensitivities in the higher risk samples including Docetaxel (target pathway: Mitosis), AZD7762 (Cell cycle), Dasatinib (kinases), Lapatinib (EGFR signaling), WIKI4 (WNT signaling), MIM1 (Apoptosis regulation), and BPD-00008900 (other). Above result may provide values in clinical individual therapy.

## 4 Discussion

In this paper, we first analyzed the transcriptome, genomic alterations, and prognostic analysis of 10 cuproptosis-related genes in LUAD, and then clustered lung cancer patients into two subgroups according to the mRNA expression of the ten cuproptosis-related genes. A prognostic model was constructed based on the differentially expressed genes between the two subgroups. Through enrichment analysis, it was found that there were differences in cell cycle, mitosis, and p53 signaling pathway between high and low-risk groups. In addition, the High-risk group samples exhibited different immune infiltration patterns compared with the low-risk group.

Seven of the ten copper death-related genes were significantly up- or down-regulated in lung adenocarcinoma tissues. The FDX1 gene is a core regulator of copper death, which induces the conversion of bivalent copper to monovalent copper, and is also an upstream regulator of proteolipidation in TCA (Rayess et al., 2012; Dörsam and Fahrer, 2016; Tsvetkov et al., 2022). FDX1 was significantly downregulated in LUAD, which may be related to the escape of cuproptosis in lung cancer cells. We found that seven of ten cuproptosis-related genes were significantly associated with prognosis according to KM survival analysis result. The above results suggest that cuproptosis has a potential role in the occurrence and prognosis of LUAD. Through SNV and CNV analysis, we found that the gene mutation frequency of CDKN2A is much higher than that of other copper death genes, and there are also significant CDKN2A homozygous and heterozygous deletions in CNV. CDKN2A is a cell cycle regulator whose loss of function is closely related to the progression, prognosis, and treatment of lung cancer (Kim et al., 2016; Jeong et al., 2018; Liu et al., 2020; Chakraborty et al., 2021; Gutiontov et al., 2021). However, how CDKN2A regulates cuproptosis in lung cancer remains to be elucidated. In addition to CDKN2A, genes, including DLD, LIPT1, GLS, PDHB, and PDHA1, also have significant CNVs, indicating the heterogeneity of lung cancer tissue.

We divided the LUAD samples into two subtypes based on the mRNA expression of 10 cuproptosis-related genes. CDKN2A, DLAT, MTF1, and PDHA1 were highly expressed in subtype 2, while PDHB was highly expressed in subtype 1. Among them, patients with subtype 2 lung cancer had a worse prognosis. The existence of two subtypes of cuproptosis in lung adenocarcinoma and the different prognoses of the two subtypes suggest that cuproptosis represents a promising investigating subject with the potential to guide the precision treatment of lung adenocarcinoma.

We established a prognostic model consisting of 22 genes using the CPDGs. The 22 genes included ANKRD29, C4BPA, CDC7, CDH17, CDKN3, CLDN2, DKK1, FOSL1, GPR37, GPRIN1, GSTA1, HJURP, HLF, KIF20A, KLK11, LPL, PRC1, RFC4, RNASEH2A, TCF19, TNNT1, and UBE2S. Among these genes, many are associated with the proliferation and invasiveness of lung cancer. C4BPA, as a cofactor of soluble complement inhibitor factor I, can help non-small cell lung cancer cells escape the cytotoxic activity of the complement system and enhance the invasiveness of tumor cells (Okroj et al., 2008). The CDC7 gene encodes a cell division cyclin with kinase activity essential for the G1/S transition. High CDC7 expression was significantly associated with p53 gain-of-function mutation status and predicted poor clinical prognosis in lung adenocarcinoma patients (Datta et al., 2017). CDKN3 may be involved in cell cycle regulation, and high expression of CDKN3 predicts poor prognosis in lung cancer patients (Hannon et al., 1994; Fan et al., 2015). CLDN2, a member of the claudin protein family, is a membrane protein localized at tight junctions, which may increase the mRNA level and enzymatic activity of MMP-9 by increasing the nuclear distribution of Sp1, and promote A549 cell migration (Ikari et al., 2011). DKK1 encodes a secreted protein that promotes the proliferation, invasion, and growth of cancer cell lines (Niehrs, 2006). FOSL1 is a transcription factor, and high expression of FOSL1 predicts poor prognosis in mutant KRAS lung cancer (Vallejo et al., 2017). GPR37 belongs to the G protein-coupled receptor family, and GPR37 can induce lung adenocarcinoma cell cycle arrest in G1 phase by binding to CDK6, thereby enhancing the progression and migration of lung adenocarcinoma cells. GPRIN1 can promote the proliferation and migration of lung cancer (Zhuang et al., 2020). GSTA1 is an enzyme that promotes the binding of glutathione to target electrophilic compounds and promotes lung cancer cell invasion and adhesion (Wang et al., 2017). HJURP is an important factor promoting the immortalization of cancer cells and is associated with poor prognosis in lung cancer patients (Kato et al., 2007). HLF can promote the distant metastasis of non-small cell lung cancer to multiple organs through the PPAR/NF-κb pathway (Chen et al., 2020). High expression of KIP20A can enhance the resistance of A549 cell line to ionizing radiation (Xiu et al., 2018). LPL encodes a protein lipase; high expression of LPL predicts poor prognosis in non-small cell lung cancer and can be highly expressed in tumor-associated macrophage subsets (Podgornik et al., 2013). As a transcriptional target gene of notch1 signaling pathway, RFC4 can promote the metastasis and stemness of non-small cell lung cancer through a positive feedback loop (Liu et al., 2021). RNASEH2A, a nucleic acid-degrading enzyme associated with cell proliferation, DNA replication, and gene instability, promotes LUAD cell proliferation and reduces apoptosis (Zhang et al., 2021). TCF19 gene can promote the proliferation of non-small cell lung cancer cells by inhibiting FOXO1 (Zhou et al., 2019). UBE2S gene encodes a member of the ubiquitin-conjugating enzyme family. High expression of UBE2S activates the NF-κB pathway and promotes the metastasis of lung adenocarcinoma (Ho et al., 2021). We calculated the risk score of each sample according to the prognostic model formula and compared the distribution of risk scores in different clinical subgroups. The results showed that the risk score increased with tumor invasion degree, lymph node metastasis level, and distant metastasis degree. Moreover, the results were in substantial agreement across the three cohorts, instructing that cuproptosis risk score and lung adenocarcinoma aggressiveness are closely related. Recently, similar studies investigating the prognostic model for lung adenocarcinoma using cuproptosis signature occurred (Li et al., 2022; Wang et al., 2022; Zhang et al., 2022). Among them, Li, et al. applied neural network to establish a cuproptosis prognostic model. Wang, et al. and Zhang, et al. constructed a cuproptosis signature using lasso method based on the 10 or 13 cuprotosis related genes, which regarded TCGA as training cohort. In our study, we applied the 169 CPGs which represented more characters to establish the prognostic model to quantify the cuproptosis related patterns. To compare the prognostic efficacy between our 22-genes signature and other two published cuproptosis-related signatures (Wang et al., 2022; Zhang et al., 2022), we calculated the C-index of prognostic models in three validation cohorts including GSE31210, GSE3141, and GSE72094. The C-index of 22-genes signature were higher than Wang, et al. and Zhang, et al. signatures in GSE31210 and GSE72094. In GSE3141, zhang, et al. signature exhibited highest C-index and 22-genes signature was in the second place (Supplementary Figure S8). Due to the low sample size in GSE3141 (58 samples), the results in GSE31210 and GSE72094 were more convincing. Overall, 22-genes signature showed a better prognostic efficacy than the others.

According to KM survival analysis and ROC curve results, the prognostic model showed good survival prediction ability in both the training and validation sets. Multivariate COX analysis showed that after adjustment for other confounding clinical factors, the risk score could still predict the prognosis of lung cancer patients well. Therefore, the risk score of the cuproptosis prognostic model is an independent prognostic predictor. In order to investigate whether the cuproptosis prognostic model combined with stage can exhibit stronger prognostic prediction ability, we drew a nomogram based on multivariate COX analysis. The calibration and timeROC curves showed that the cuproptosis prognostic model combined with the stage had a better OS prediction effect. This suggests that the cuproptosis prognostic model can optimize the predictive power of stage for prognosis in lung cancer patients.

To understand the underlying molecular mechanism of copper death regulating lung adenocarcinoma, we performed GO and KEGG enrichment analyses for differentially expressed genes between high- and low-risk groups. The results showed significant differences in cell cycle, mitosis, and p53 signaling pathway between high- and low-risk groups. In the study of AML, glioblastoma, and non-small cell lung cancer, copper ion-binding small molecule compounds or drugs can induce oxidative stress and cell cycle arrest in tumor cells (Duan et al., 2014; Hassani et al., 2018; Shimada et al., 2018). Meanwhile, in the colon adenocarcinoma cell line HCT116, copper ions can reduce the induction of the P53 pathway by cisplatin in cancer cells (Kabolizadeh et al., 2007). These pieces of evidence suggest that copper metabolism is involved in tumor suppression *via* regulating the cell cycle and p53 pathway, which is consistent with our enrichment analysis results and points out the direction for further cuproptosis investigations.

The immune microenvironment plays an integral role in tumor initiation and progression. To this end, we analyzed immune infiltration in the high- and low-risk groups. Activated dendritic cells, mast cell enrichment fraction, and type 2 interferon response levels were significantly down-regulated in the high-risk group, while the proportions of neutrophils and M0 macrophages also showed a significant upward trend in the high-risk group. Mature dendritic cells promote T cell activation in tertiary lymphoid structures and explain the high expression of CD8^+^ T cells and more prolonged overall survival in some lung cancer patients (Goc et al., 2014). Mast cells predicting prognosis in patients with lung adenocarcinoma may depend on the microlocalization of mast cell infiltration, and high-density mast cell infiltration in non-small cell lung cancer epithelial cells suggests a better prognosis (Welsh et al., 2005). IFN-γ can inhibit the proliferation of lung adenocarcinoma cells by activating the JAK2-STAT1 pathway, and blocking the PI3K-AKT pathway can enhance the anti-proliferation ability of IFN-γ (Gao et al., 2018). Neutrophils promote the entire carcinogenesis process, including carcinogenesis, proliferation, and metastasis. And in targeted therapy for metastatic renal cell carcinoma, increased neutrophil counts predict a poor prognosis (Ocana et al., 2017). An increased proportion of M0 macrophages is associated with poor prognosis in lung cancer patients (Liu et al., 2017). The above evidence indicates that the changes mentioned above in the level of immune cell infiltration and the enrichment of immune responses account for the poor prognosis of the high-risk group. The IPS is an indicator for evaluating the immunogenicity of tumor samples, and higher IPS predicts higher sensitivity to immunotherapy (Charoentong et al., 2017). A comparison of IPS between high- and low-risk groups found that the low-risk group had higher IPS, indicating that patients in the low-risk group were more likely to benefit from immunotherapy. In addition, Chemotherapy remains the major treatment modality for cancers. We obtained 61 agents whose drug sensitivities were associated with risk scores. Seven kinds of drugs showed higher sensitivities in higher risk score samples were identified, which including Docetaxel, AZD7762, Dasatinib, Lapatinib, WIKI4, MIM1, and BPD-00008900. Agents including Dasatinib (Haura et al., 2010), Docetaxel (Joshi et al., 2014), Lapatinib (Blumenschein et al., 2010) were effective agent in treatment of LUAD, which may exhibit better curative effect in LUAD patients with higher risk scores. Therefore, the risk score may represent a guiding index for the precise treatment of lung cancer patients.

There are still shortcomings in this study. Although the prognosis predictive ability of the prognostic model was validated in multiple cohorts, the data were derived from a public database of retrospective studies. Its predictive ability remains to be verified in randomized clinical trials. IPS can estimate the sensitivity of lung cancer patients to immunotherapy but cannot substitute for the real treatment response.

In this paper, lung adenocarcinoma patients were clustered into two subtypes according to the expression levels of 10 copper death genes, and the differentially expressed genes related to prognosis between the two subtypes were obtained through differential analysis and survival analysis, and the cuproptosis prognostic model was constructed using cox lasso regression analysis, we found that the risk score was a good predictor of overall survival in patients with lung adenocarcinoma. In addition, there were different immune infiltration patterns between high- and low-risk groups, and the low-risk group was more sensitive to immunotherapy, according to IPS. LUAD patients with higher risk scores may benefit from seven kinds of chemotherapy drugs according to the drug sensitivities analysis. Through the above analysis, we found an intimate relationship between cuproptosis and lung adenocarcinoma occur, prognosis and treatment, and the cuproptosis prognostic model may be valuable for prognosis prediction and immunotherapy guidance in lung adenocarcinoma patients.

## Data Availability

The original contributions presented in the study are included in the article/Supplementary Material, further inquiries can be directed to the corresponding author.

## Supplementary material

Supplementary Figure S1 The gene expression difference between lung cancer and paired normal tissues in GSE32863 and TCGA. **(A)**The boxplot showed the 10 cuproptosis genes expression pattern in GSE32863 cohort. **(B)** The violinplot showed the prognostic signature genes expression pattern in TCGA cohort. **(C)** The boxplot showed the prognostic signature genes expression pattern in GSE32863 cohort. The symbols *, **, ***, *** corresponded to *p* < 0.05, 0.01, 0.001, 0.0001, respectively *p* < 0.05 was regarded as significant.

Supplementary Figure S2 Seven cuproptosis-related genes correlated with prognosis significantly. PDHA1, DLAT, DLD, and CDKN2A were high-risk factors for the prognosis of LUAD patients in the TCGA cohort, while MTF1, LIPT1, and GLS were favorable factors.

Supplementary Figure S3 Heatmap for 22 prognostic model genes and risk scores distribution among LUAD patients in three validation sets. **(A,D)** were for GSE72094, **(B,E)** were for GSE31210, **(C,F)** were for GSE3141.

Supplementary Figure S4 The distribution of risk scores in different clinical features in the GSE31210 cohort **(A–C)** and GSE72094 cohort **(D–F)**.

Supplementary Figure S5 GO and KEGG enrichment results for three validation cohorts. **(A–C)** were the GO enrichment results; **(D–F)** were the KEGG results. **(A,D)** were for GSE72094, **(B,E)** were for GSE31210, **(C,F)** were for GSE3141.

Supplementary Figure S6 immune cell and function signatures were compared between the high and low-risk groups in three validation sets. **(A,D)** were for GSE72094, **(B,E)** were for GSE31210, **(C,F)** were for GSE3141.

Supplementary Figure S7 CIRBERSORT, ESTIMATE and IPS results were compared between the high and low-risk groups. **(A,D)** were for GSE72094, **(B,E)** were for GSE31210, **(C,F)** were for GSE3141. **(G–I)** were the ESTIMATE results (from left to right: ESTIMATE score, immune score, stromal score, and tumor purity) in the merge cohort.

Supplementary Figure S8 The forest plot showed the C-index differences among the 22-genes signature, Zhang, et al, and Wang, et al in three validation cohorts.
