# Supplementary figures and images for: Identification of two molecular subtypes and a novel prognostic model of lung adenocarcinoma based on a cuproptosis-associated gene signature
Source: Front Genet. 2023 Jan 12;13:1039983. doi: 10.3389/fgene.2022.1039983 (PMC9877306; doi:10.3389/fgene.2022.1039983)

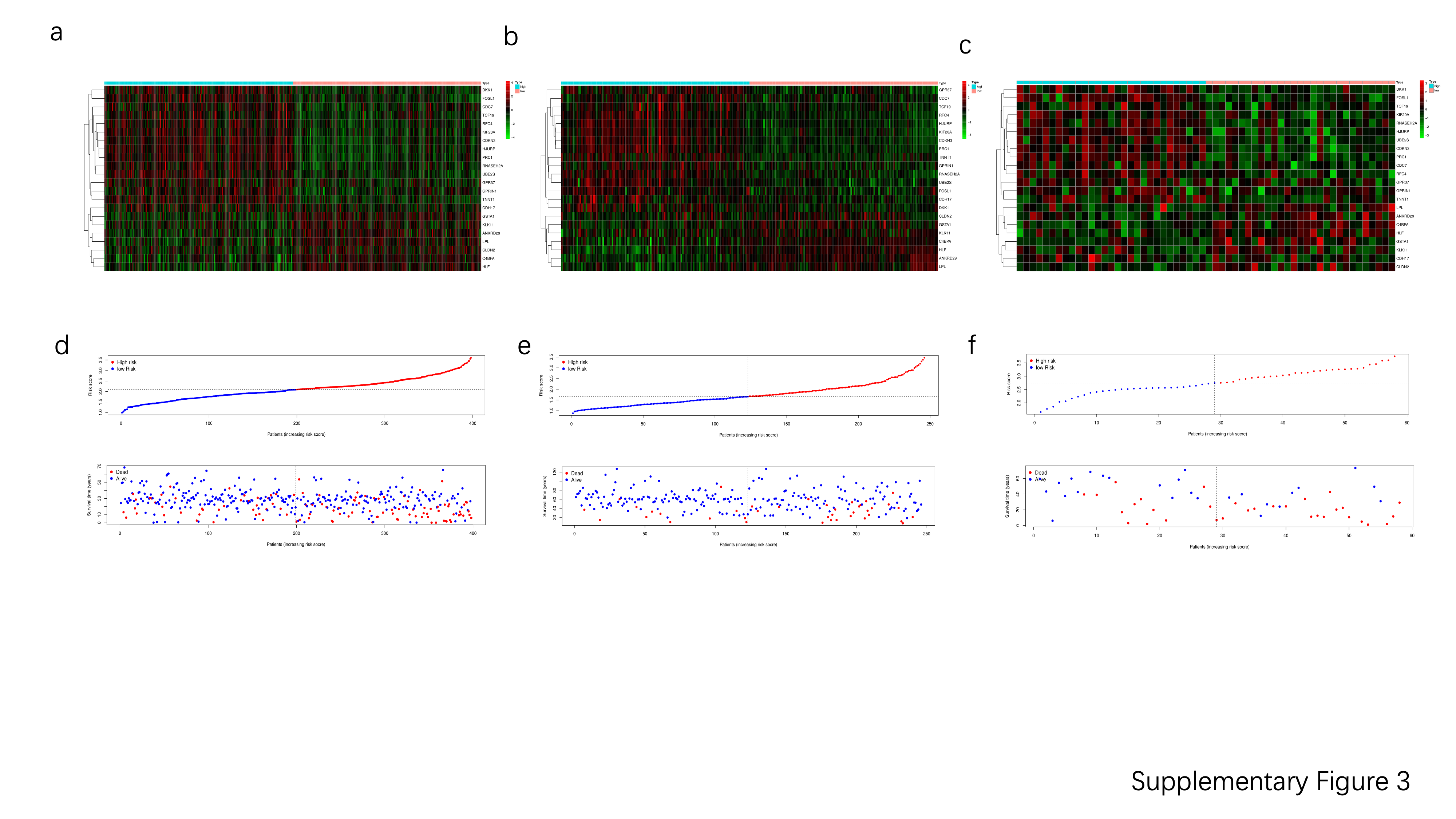

Supplement: Supplementary file 1 [file Image3.TIFF]

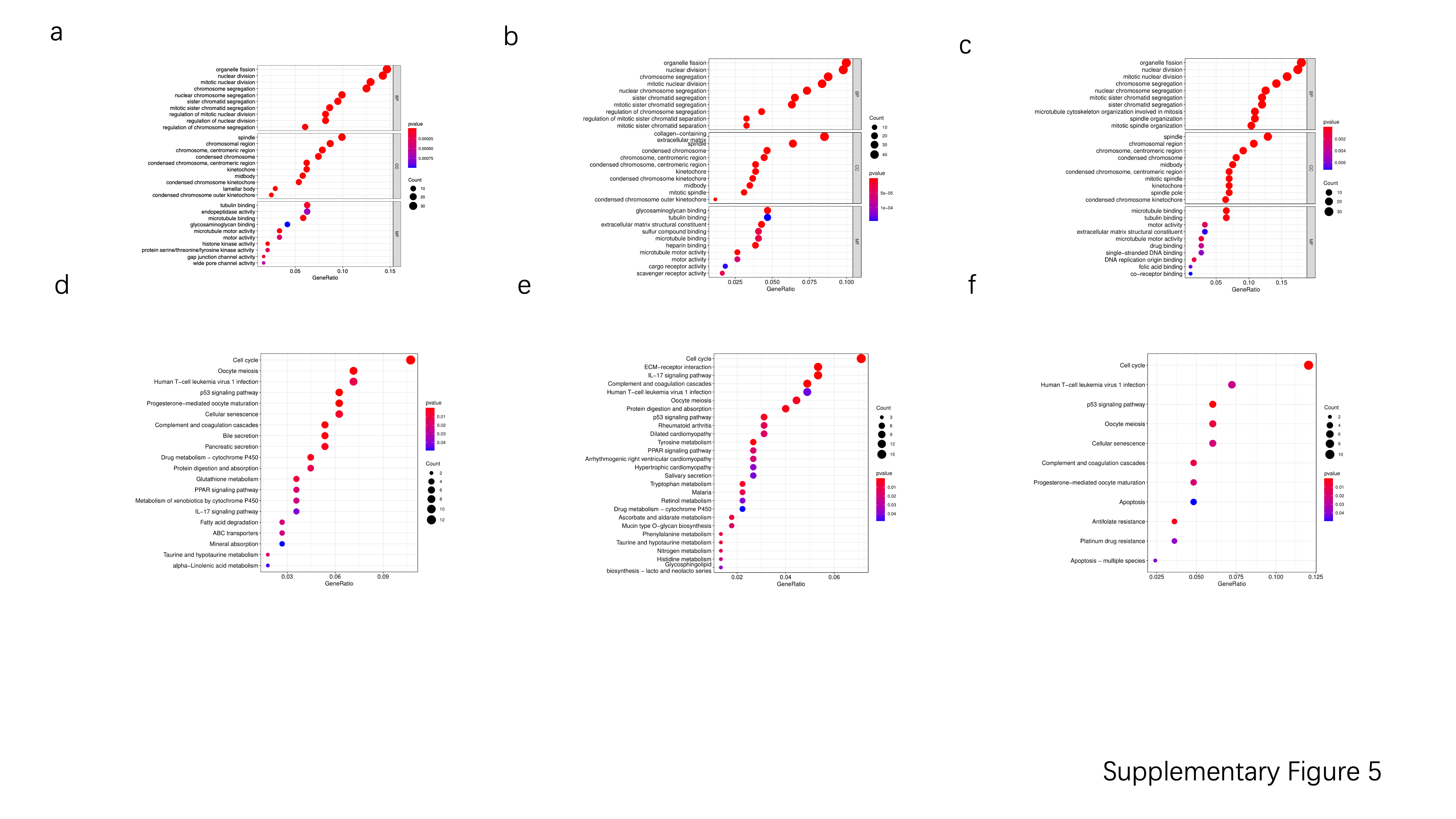

Supplement: Supplementary file 2 [file Image5.TIFF]

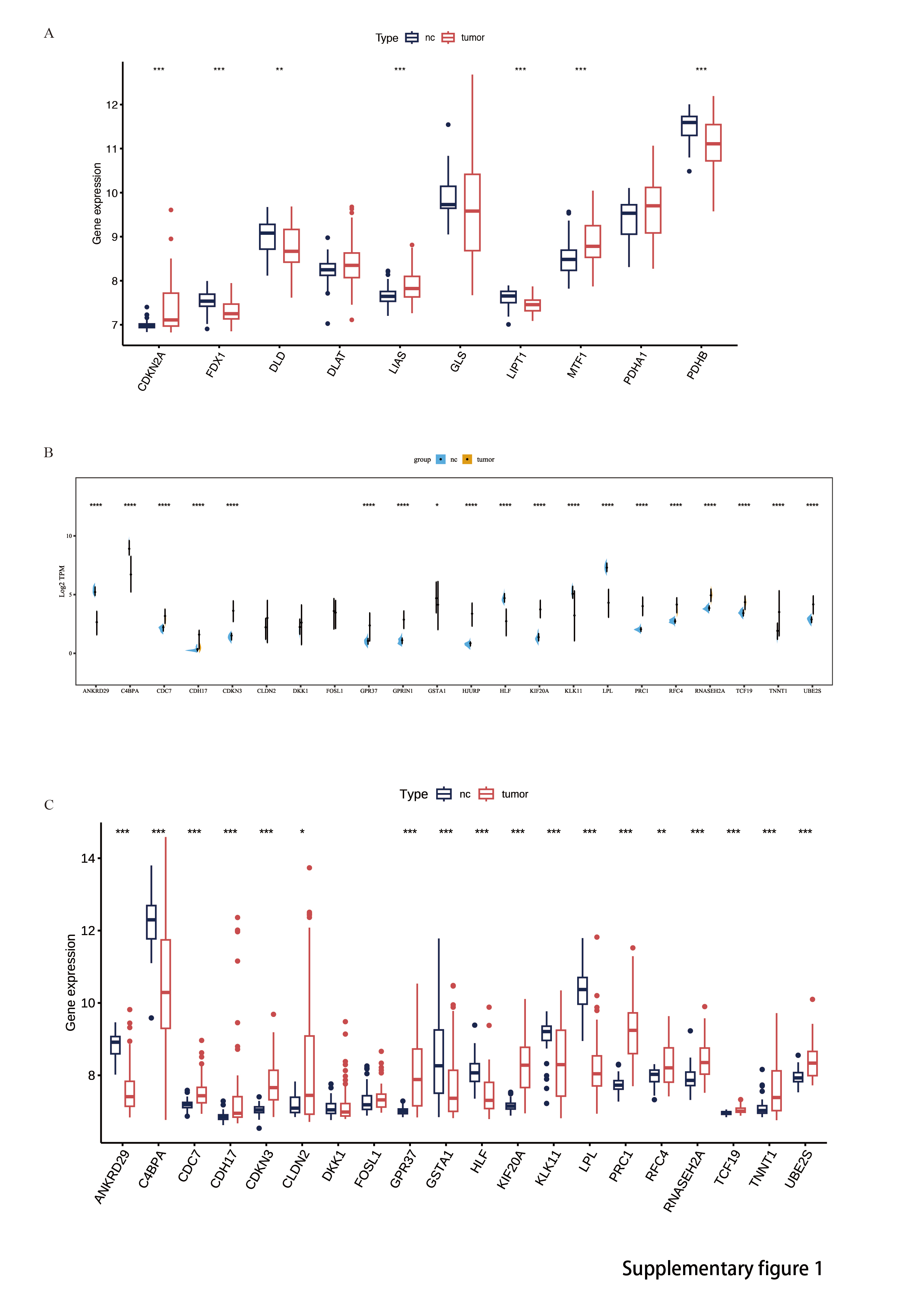

Supplement: Supplementary file 3 [file Image1.JPEG]

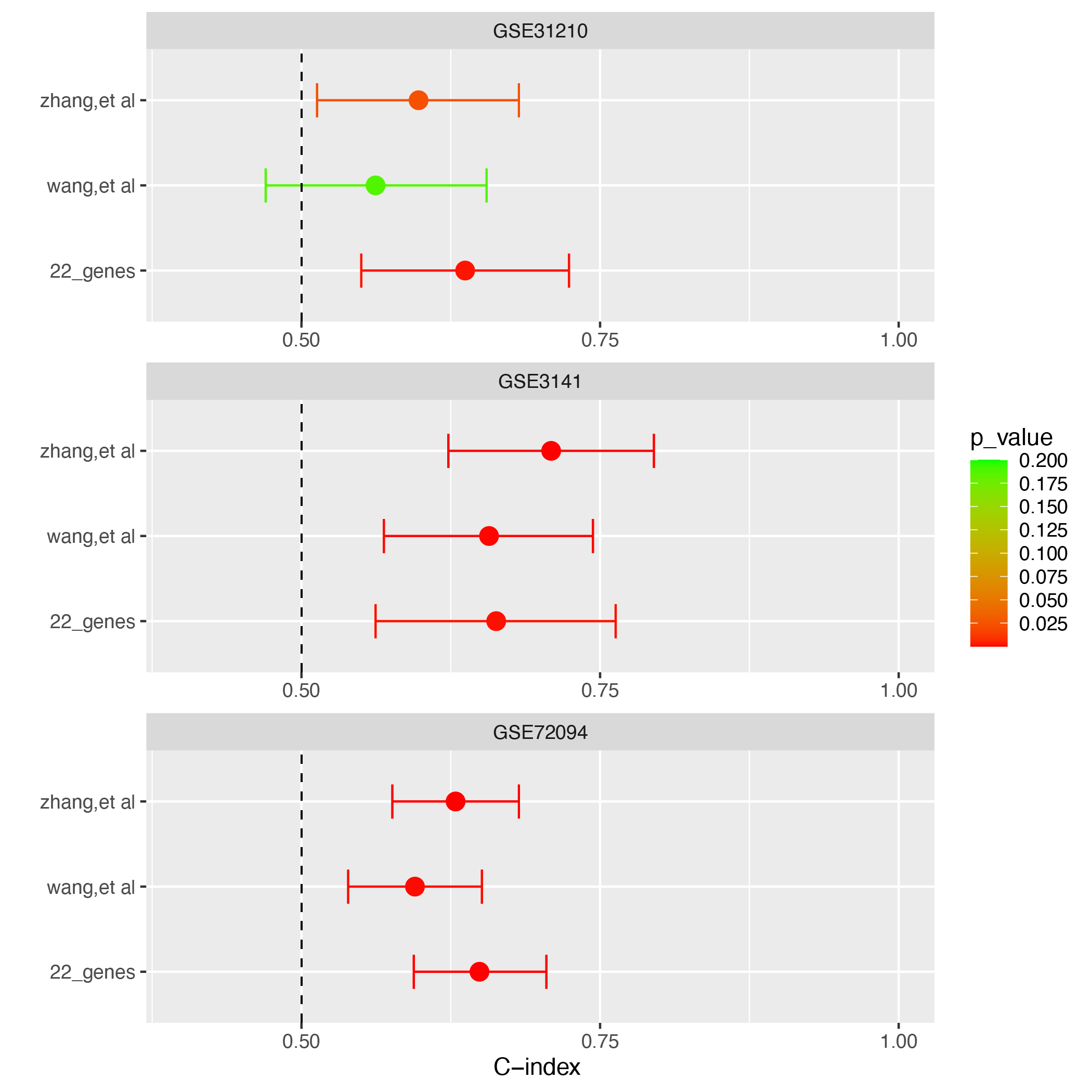

Supplement: Supplementary file 5 [file Image8.TIF]

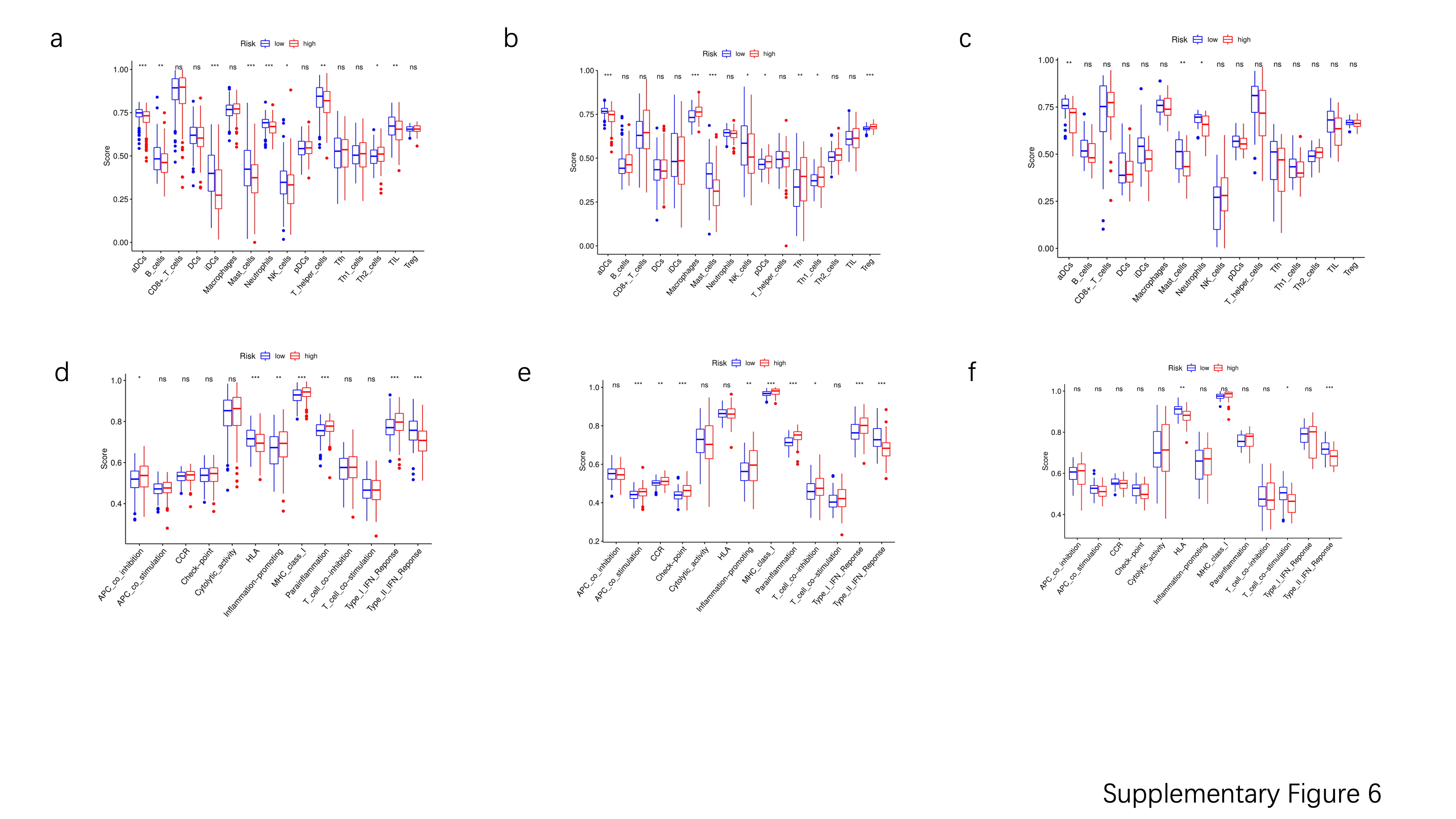

Supplement: Supplementary file 6 [file Image6.TIFF]

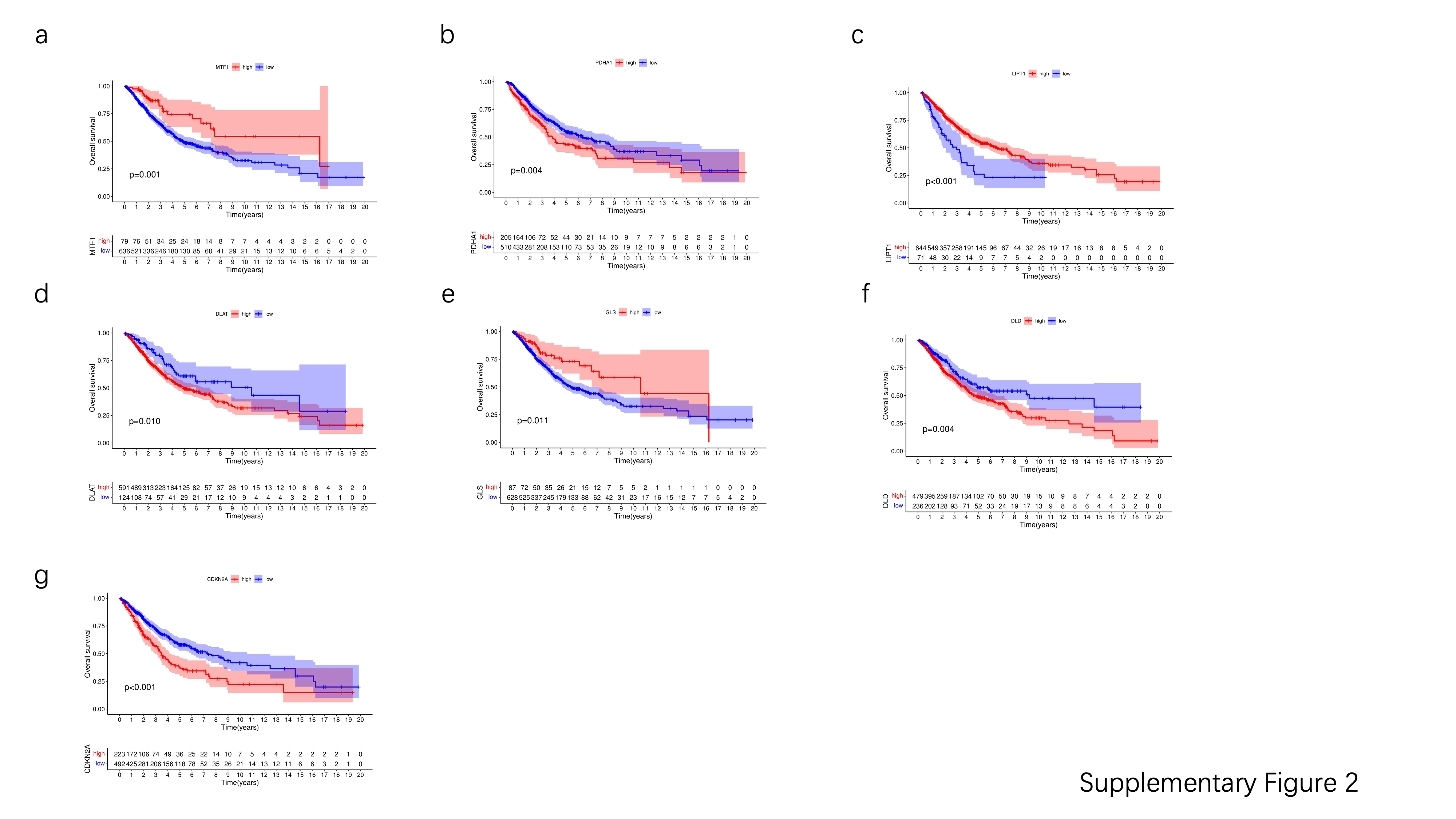

Supplement: Supplementary file 7 [file Image2.TIFF]

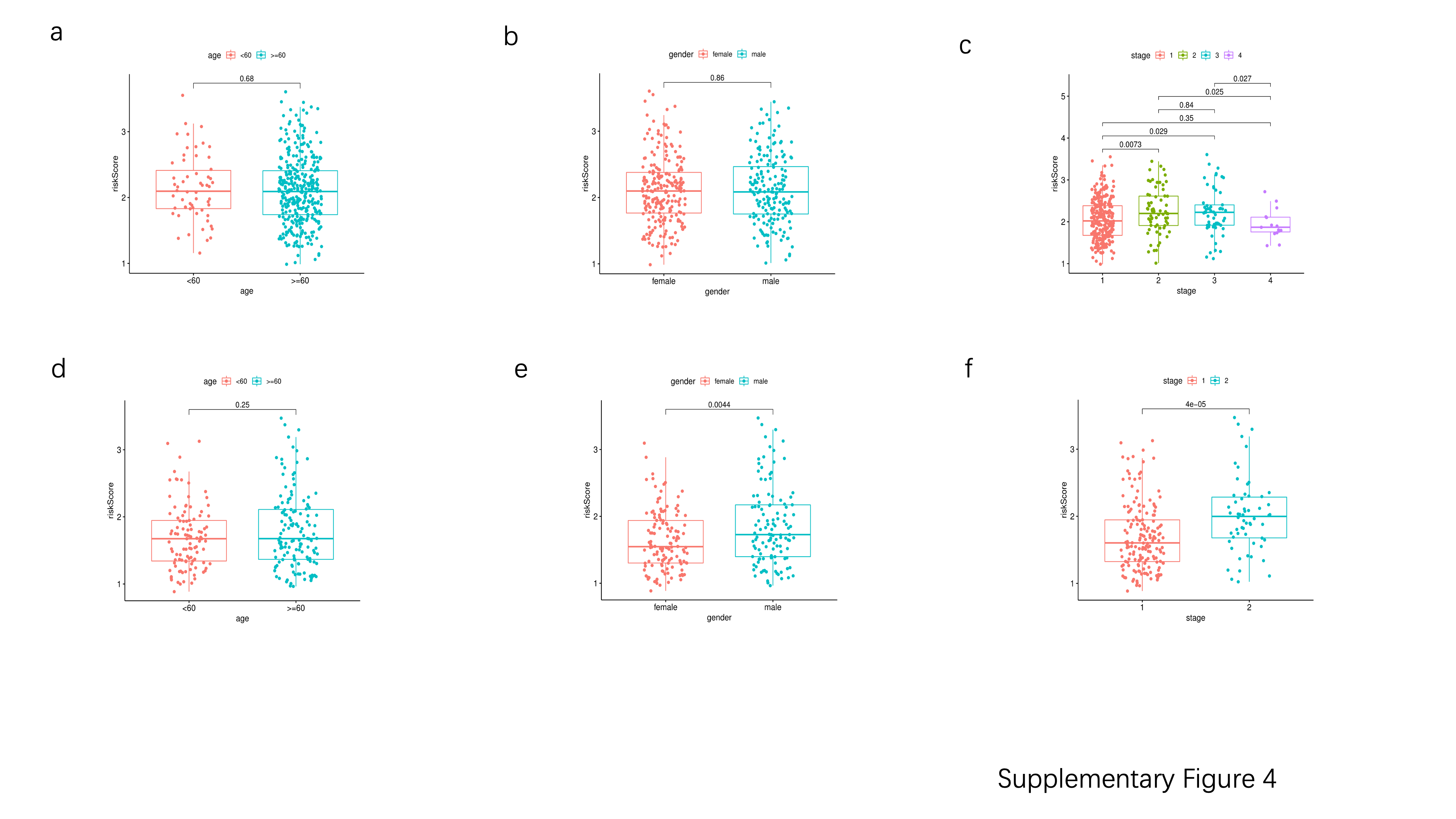

Supplement: Supplementary file 8 [file Image4.TIFF]

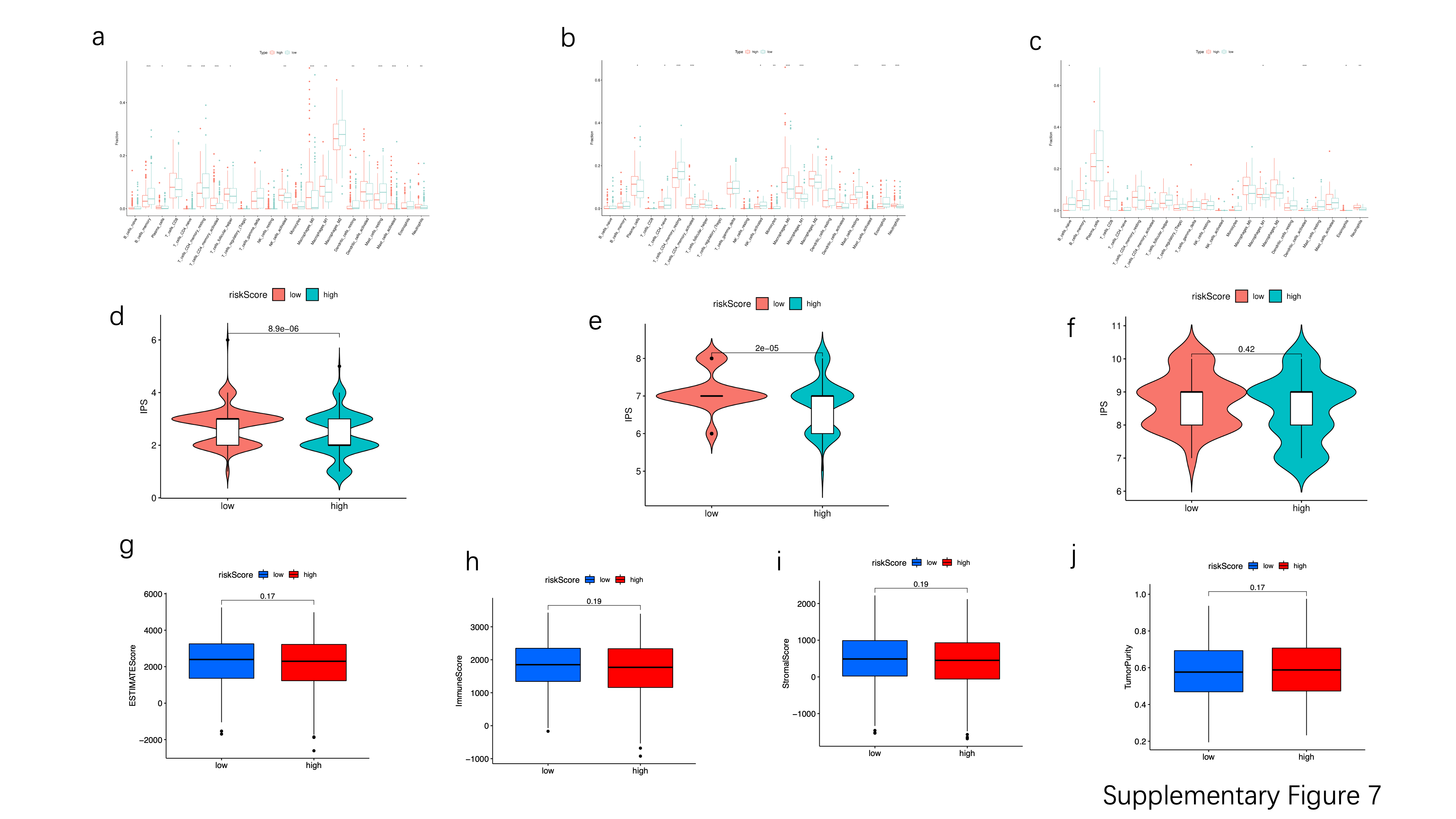

Supplement: Supplementary file 9 [file Image7.TIFF]
